# Supplementary material for: Structural and functional brain asymmetry in relation to heterogeneous causes of situs inversus totalis
Source: Brain Struct Funct. 2026 Mar 13;231(3):38. doi: 10.1007/s00429-026-03098-5 (PMC12987916; doi:10.1007/s00429-026-03098-5)
Supplement: Supplementary file 1 — Supplementary Material 1 [file 429_2026_3098_MOESM1_ESM.docx]

**Supplementary materials for *Wang et al., Structural and functional brain asymmetry in relation to heterogeneous causes of situs inversus totalis***

To evaluate whether the presence of first-degree related pairs (SIT003-SIT066; SIT064-SIT065) influenced the results, all analyses were repeated after excluding one individual from each related pair, in all four possible combinations as detailed below. These sensitivity analyses were performed for functional brain asymmetry, atypical lateralization patterns and handedness, as well as structural brain asymmetry measures.

**Sensitivity analysis on functional brain asymmetry**

**Excluding SIT003 and SIT064**, no significant effects of group were observed for word generation (F(2, 69) = 2.56, p_adjusted= 0.25, η²_partial = 0.07), praxis (F(2, 69) = 0.64, p_adjusted= 0.53, η²_partial = 0.02), spatial attention (F(2, 69) = 4.43, p_adjusted= 0.06, η²_partial = 0.11), or face recognition (F(2, 69) = 1.75, p_adjusted= 0.36, η²_partial = 0.05).

**Excluding SIT003 and SIT065**, no significant effects of group were observed for word generation (F(2, 69) = 2.44, p_adjusted= 0.28, η²_partial = 0.07), praxis (F(2, 69) = 0.83, p_adjusted= 0.54, η²_partial = 0.02), spatial attention (F(2, 69) = 4.52, p_adjusted= 0.06, η²_partial = 0.12), or face recognition (F(2, 69) = 1.33, p_adjusted= 0.54, η²_partial = 0.04).

**Excluding SIT066 and SIT064**, similarly, no significant group effects were detected for word generation (F(2, 69) = 2.94, p_adjusted= 0.18, η²_partial = 0.08), praxis (F(2, 69) = 1.10, p_adjusted= 0.34, η²_partial = 0.03), spatial attention (F(2, 69) = 4.34, p_adjusted= 0.07, η²_partial = 0.11), or face recognition (F(2,69) = 1.84, p_adjusted= 0.33, η²_partial = 0.05).

**Excluding SIT066 and SIT065**, similarly, no significant group effects were detected for word generation (F(2, 69) = 2.80, p_adjusted= 0.20, η²_partial = 0.08), praxis (F(2, 69) = 1.31, p_adjusted= 0.50, η²_partial = 0.04), spatial attention (F(2, 69) = 4.44, p_adjusted= 0.06, η²_partial = 0.11), or face recognition (F(2,69) = 1.42, p_adjusted= 0.50, η²_partial = 0.04).

**Sensitivity analysis on atypical functional laterality patterns and handedness**

**Excluding SIT003 and SIT064**, there was no significant difference in variance of the number of atypically lateralized functions across genetically solved, unsolved and control groups (Levene’s test, p = .14). The rate of left-handedness did not differ between genetically solved and unsolved SIT groups (Fisher’s exact p = 1).

**Excluding SIT003 and SIT065**, there was no significant difference in variance of the number of atypically lateralized functions across genetically solved, unsolved and control groups (Levene’s test, p = .15). The rate of left-handedness did not differ between genetically solved and unsolved SIT groups (Fisher’s exact p = 1).

**Excluding SIT066 and SIT064**, Levene’s test indicated a significant difference in variance across groups (p = .03). Follow-up pairwise variance comparisons showed that variability in the number of atypically lateralized functions was greater in genetically solved SIT cases than in controls (*p* = 0.04), but variance did not differ significantly between the solved and unsolved SIT groups (*p* = 0.32), nor between the unsolved SIT group and the controls (*p* = 0.20). Handedness rates again did not differ between genetically solved and unsolved SIT groups (Fisher’s exact p = 1).

**Excluding SIT066 and SIT064**, Levene’s test indicated a significant difference in variance across groups (p = .03). Follow-up pairwise variance comparisons showed that variability in the number of atypically lateralized functions was greater in genetically solved SIT cases than in controls (*p* = 0.04), but variance did not differ significantly between the solved and unsolved SIT groups (*p* = 0.33), nor between the unsolved SIT group and the controls (*p* = 0.18). Handedness rates again did not differ between genetically solved and unsolved SIT groups (Fisher’s exact p = 1).

**Sensitivity analysis on structural asymmetry measures:**

**Petalia and bending**

**Excluding SIT003 and SIT064**, significant associations between the three-level group factor (genetically solved SIT cases, unsolved SIT cases, and controls) and frontal petalia (F(2, 69) = 4.53, p_adjusted= .03, η²_partial= 0.12), occipital petalia (F(2, 69) = 7.85, p_adjusted= .003, η²_partial= 0.18), and occipital bending (F(2, 69) = 9.74, p_adjusted< .001, η²_partial= 0.22) were observed, whereas frontal bending was not significant (F(2, 69) = 0.87, p = .42, η²_partial= 0.02). Post hoc comparisons indicated that these effects were driven by differences between solved SIT cases and controls, as well as between unsolved SIT cases and controls, with no significant differences between solved and unsolved SIT cases across any torque measure (all p ≥ .60).

**Excluding SIT003 and SIT065**, significant associations between the three-level group factor (genetically solved SIT cases, unsolved SIT cases, and controls) and frontal petalia (F(2, 69) = 4.29, p_adjusted= .04, η²_partial= 0.11), occipital petalia (F(2, 69) = 7.61, p_adjusted= .003, η²_partial= 0.18), and occipital bending (F(2, 69) = 9.70, p_adjusted< .001, η²_partial= 0.22) were observed, whereas frontal bending was not significant (F(2, 69) = 1.02, p = .37, η²_partial= 0.03). Post hoc comparisons indicated that these effects were driven by differences between solved SIT cases and controls, as well as between unsolved SIT cases and controls, with no significant differences between solved and unsolved SIT cases across any torque measure (all p ≥ .60).

**Excluding SIT066 and SIT064**, a highly similar pattern was observed. Significant group effects were again found for frontal petalia (F(2, 69) = 4.16, p_adjusted= .04, η²_partial= 0.11), occipital petalia (F(2, 69) = 7.76, p_adjusted= .003, η²_partial= 0.18), and occipital bending (F(2, 69) = 9.93, p_adjusted< .001, η²_partial= 0.22), while frontal bending again showed no significant group effect (F(2, 69) = 0.87, p = .42, η²_partial= 0.02). As in the primary analysis, post hoc comparisons demonstrated that significant omnibus effects were driven by differences between SIT cases and controls, and not by differences between genetically solved and unsolved SIT cases (all p ≥ .58).

**Excluding SIT066 and SIT065**, a highly similar pattern was observed. Significant group effects were again found for frontal petalia (F(2, 69) = 3.93, p_adjusted= .048, η²_partial= 0.10), occipital petalia (F(2, 69) = 7.53, p_adjusted= .003, η²_partial= 0.18), and occipital bending (F(2, 69) = 9.89, p_adjusted< .001, η²_partial= 0.22), while frontal bending again showed no significant group effect (F(2, 69) = 1.03, p = .36, η²_partial= 0.03). As in the primary analysis, post hoc comparisons demonstrated that significant omnibus effects were driven by differences between SIT cases and controls, and not by differences between genetically solved and unsolved SIT cases (all p ≥ .58).

**Transverse sinus**

**Excluding SIT003 and SIT064**, significant associations between group and transverse sinus volumes were again observed for both the left transverse sinus (F(2, 69) = 4.49, p_adjusted= .03, η²_partial= 0.11) and the right transverse sinus (F(2, 69) = 4.28, p_adjusted= .03, η²_partial= 0.11). Post hoc comparisons revealed no significant differences between solved and unsolved SIT cases for either the left (*p* = .93) or right (*p* = .87) transverse sinus volumes. The significant omnibus effects were again driven by differences between SIT cases and controls.

**Excluding SIT003 and SIT065**, significant associations between group and transverse sinus volumes were again observed for both the left transverse sinus (F(2, 69) = 4.12, p_adjusted= .03, η²_partial= 0.10) and the right transverse sinus (F(2, 69) = 4.35, p_adjusted= .03, η²_partial= 0.11). Post hoc comparisons revealed no significant differences between solved and unsolved SIT cases for either the left (*p* = .97) or right (*p* = .91) transverse sinus volumes. The significant omnibus effects were again driven by differences between SIT cases and controls.

**Excluding SIT066 and SIT064**, similar results were obtained, with significant group effects for both left (F(2, 69) = 4.33, p_adjusted= .03, η²_partial= 0.11) and right (F(2, 69) = 4.38, p_adjusted= .03, η²_partial= 0.11) transverse sinus volumes. As in the primary analysis, no significant differences were observed between solved and unsolved SIT groups in post hoc comparisons (all p > .85), indicating that the observed effects were driven by differences between SIT cases and controls rather than by causal heterogeneity within the SIT group.

**Excluding SIT066 and SIT065**, similar results were obtained, with significant group effects for both left (F(2, 69) = 3.97, p_adjusted= .03, η²_partial= 0.10) and right (F(2, 69) = 4.44, p_adjusted= .03, η²_partial= 0.11) transverse sinus volumes. As in the primary analysis, no significant differences were observed between solved and unsolved SIT groups in post hoc comparisons (all p > .85), indicating that the observed effects were driven by differences between SIT cases and controls rather than by causal heterogeneity within the SIT group.

**Overall summary of sensitivity analyses**

Sensitivity analysis showed that the results of the primary analysis were barely affected by the presence of two pairs of siblings in the dataset. The exclusion of SIT066 resulted in there being a significant effect in the Levene test of equal variances for the number of atypically lateralized brain functions across all three groups together (solved SIT, unsolved SIT, control). However, the direct pairwise group comparison of solved SIT versus unsolved SIT cases found no significant difference. The conclusions from the primary analysis remain unaltered.
